# Assessing the Oncological Safety of Glucagon-Like Peptide-1 Receptor Agonists: A Systematic Review and Meta-Analysis

**DOI:** 10.7759/cureus.96071

**Published:** 2025-11-04

**Authors:** Jaber H Jaradat, Rawan I Al-Ahmad, Maha Al Jaghbeer, Ahlam A Abdelaziz, Nour M Abu Afifeh, Ghazi Abu Afifeh, Asad Rao, Amjad Alzoubi, Abdulqadir J Nashwan

**Affiliations:** 1 School of Medicine, Mutah University, Al Karak, JOR; 2 Faculty of Medicine, Yarmouk University, Irbid, JOR; 3 Administrative and Financial Affairs, Jordan Food and Drug Administration, Amman, JOR; 4 Faculty of Medicine, University of Jordan, Amman, JOR; 5 Internal Medicine, University of Jordan, Amman, JOR; 6 Medicine, Dow University of Health Sciences, Karachi, PAK; 7 Faculty of Medicine, Jordan University of Science and Technology, Irbid, JOR; 8 Nursing and Midwifery Research, Hamad Medical Corporation, Doha, QAT

**Keywords:** cancer risk, glp-1 receptor agonists, liraglutide, obesity, semaglutide

## Abstract

Glucagon-like peptide-1 (GLP-1) receptor agonists are essential for treating type 2 diabetes and promoting weight loss. Despite their therapeutic benefits, concerns have arisen regarding their potential association with pancreatic and thyroid cancers. This systematic review and meta-analysis examined the correlation between GLP-1 receptor agonists and cancer incidence in obese/overweight individuals, including both patients with diabetes and overweight/obese non-diabetic participants. A systematic search of PubMed, Scopus, and Cochrane databases identified randomized clinical trials (RCTs) for inclusion. Data extraction and risk of bias assessment followed rigorous methodologies, using the Risk of Bias 2 tool. Of the 1,882 identified studies, nine RCTs (9,078 participants) met the inclusion criteria. The studies varied in duration (12-104 weeks) and demographics, with a mean participant age of 46.9 years and a mean body mass index of 36.9 kg/m². In non-diabetic overweight/obese participants, GLP-1 receptor agonists significantly reduced body weight and HbA1c levels compared to placebo. However, varying incidences of neoplasms were observed, with liraglutide showing a statistically significant odds ratio of 2.8150 for cancer risk. Semaglutide trials have reported mixed results, with some studies showing an increase in neoplasm events in the intervention groups. Although GLP-1 receptor agonists effectively manage weight and glycemic control in overweight/obese patients, their association with increased cancer risk warrants cautious application, especially in individuals with a predisposition to thyroid or pancreatic cancers. Further studies are needed to conclusively determine the safety profile of these therapies.

## Introduction and background

Glucagon-like peptide-1 (GLP-1) is a critical incretin hormone secreted after meals in healthy individuals. It plays a vital role in glucose homeostasis by enhancing insulin secretion, suppressing glucagon release, slowing gastric emptying, reducing appetite, and potentially preventing β-cell apoptosis [1]. These effects have made GLP-1 receptor agonists (GLP-1RAs) a crucial therapeutic class for managing type 2 diabetes mellitus (T2DM). GLP-1RAs are categorized into short-acting agents (e.g., exenatide, lixisenatide) and long-acting formulations (e.g., albiglutide, dulaglutide, exenatide extended-release, and liraglutide). The short-acting agents primarily affect postprandial glucose levels by inhibiting gastric emptying. In contrast, the long-acting agents have a greater influence on fasting glucose levels due to prolonged insulinotropic and glucagonostatic actions [2].

GLP-1RAs have shown significant efficacy in improving glycemic control and promoting weight loss in diabetic and non-diabetic individuals. Clinical trials, such as SUSTAIN-6 and LEADER, have demonstrated the metabolic benefits of semaglutide. The LEAD-1 trial and comparative studies involving glimepiride and liraglutide have shown similar effectiveness in treating T2DM [3,4]. Beyond glycemic management, GLP-1RAs offer additional benefits, including improved insulin sensitivity, reduced blood pressure, and protection against cardiovascular and renal disease [5].

Despite their therapeutic advantages, GLP-1RAs have raised concerns regarding potential risks, particularly for certain cancers. Animal studies and pharmacovigilance database reviews have indicated possible associations between GLP-1RA use and thyroid or pancreatic cancers [6]. These concerns are not consistently supported across all studies; some real-world data and clinical observations have failed to find a statistically significant association, suggesting that the cancer risk remains uncertain. The inconsistency highlights the importance of incorporating real-world evidence, which offers broader insights into clinical outcomes across diverse populations and care settings.

Nevertheless, concerns persist about the risk of adverse effects such as pancreatitis, C-cell adenomas, and carcinomas with GLP-1RA use [7-9]. While current findings do not conclusively establish a carcinogenic effect, the potential link warrants further scrutiny. Particular attention has been paid to medullary thyroid cancer (MTC), as increased β-cell mass in rodent models has been paralleled by higher rates of MTC in some animal studies [9-12]. Historical literature and safety database analyses suggest GLP-1RAs may stimulate pre-existing premalignant lesions rather than cause de novo tumorigenesis [12]. However, interspecies variation in GLP-1 receptor expression complicates interpretation.

There is also emerging evidence of possible protective effects of GLP-1 on other malignancies, such as colon and breast cancers. Nevertheless, the U.S. Food and Drug Administration advises caution, and GLP-1RAs are contraindicated in individuals with personal or family histories of MTC or multiple endocrine neoplasia type 2 [13]. This highlights the importance of individual risk stratification in clinical decision-making.

Given the growing use of GLP-1RAs in treating both T2DM and obesity, this meta-analysis aims to explore their association with cancer risk in both diabetic and overweight/obese non-diabetic patients. Additionally, examining cancer incidence in users of GLP-1RA compared to those receiving a placebo sheds light on potential oncologic risks, offering essential insights into the safety of these treatments. Such investigations are necessary for balancing the clear metabolic benefits of GLP-1RAs with their long-term safety, ensuring informed, evidence-based clinical practice.

## Review

Methodology

Search Strategy

On April 20, 2024, two independent researchers searched PubMed, Scopus, and Cochrane databases. The search strategy employed Medical Subject Headings (MeSH) terms and relevant keywords, filters, and synonyms to ensure comprehensive coverage. The search approach was peer-reviewed according to the PRESS (Peer Review for Electronic Search Strategies) guidelines [14]. The search terms included were (“GLP-1 receptor agonist” OR “glucagon-like peptide-1 receptor agonist” OR “GLP-1R receptor” OR “GLP1R protein” OR “GLP-1 receptor” OR “GLP1R receptor”) AND (“glucagon-like peptide 1 receptor agonists” OR “incretin mimetics” OR “GLP-1 analogs” OR “liraglutide” OR “victoza” OR “saxenda” OR “semaglutide” OR “ozempic” OR “rybelsus” OR “exenatide” OR “byetta” OR “bydureon” OR “dulaglutide” OR “trulicity” OR “albiglutide” OR “tanzeum” OR “lixisenatide” OR “adlyxin” OR “lyxumia”) AND (“tumor” OR “neoplasm” OR “neoplasia” OR “cancer” OR “malignant neoplasm” OR “malignancy” OR “benign neoplasm” OR “benign neoplasms”). 

Additionally, the reference lists of the included studies were manually checked, and backward citation analysis was conducted. The authors were contacted via email to request missing data or provide access to the full text of their work. The PROSPERO database was also searched using the terms (“GLP-1 Receptor Agonists”) AND (“Neoplasms”) to identify ongoing studies. It was registered on the PROSPERO database, with a reference number of CRD42024553076. There were no time restrictions, and only English-language articles were considered for inclusion.

Study Selection Criteria

The meta-analysis included original randomized clinical trials (RCTs). The exclusion criteria were cross-sectional, retrospective, prospective, cohort, case reports/series, review articles, posters, commentaries, protocols, editorials, conference abstracts, and other publications without primary data. Relevant participants included individuals of any age, sex, or ethnicity who used GLP-1RAs versus a placebo.

Screening and Data Extraction

The retrieved studies were exported to Rayyan.ai, an online tool for duplicate identification and removal [15-23]. Two independent authors screened titles, abstracts, and keywords to assess their eligibility. Publications meeting the initial criteria underwent a full-text review for final inclusion. Discrepancies were resolved by consensus or a third reviewer. Data extraction was performed independently by five reviewers using a standardized spreadsheet, capturing details on study characteristics (author, year, country, and design), participant characteristics (sample size, comorbidities), intervention/exposure, outcomes, and findings related to neoplasm risk. Due to the heterogeneity of the studies, a narrative synthesis of the findings was performed, and the data were summarized in a tabular format highlighting neoplasm risk factors.

Quality Assessment and Risk of Bias

**Figure 1:**
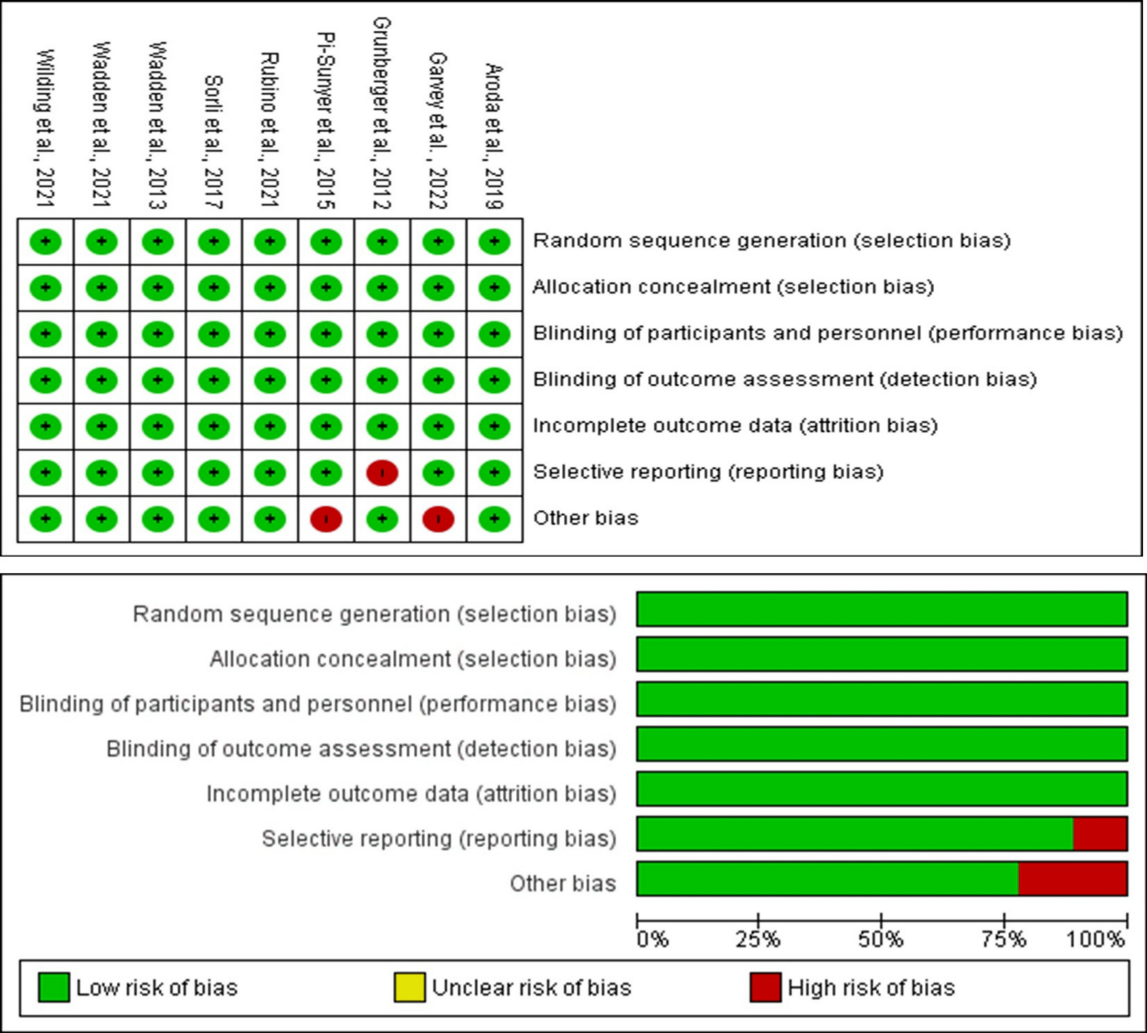
Quality assessment of the included studies using the Risk of Bias 2 tool. References: Wilding et al., 2021 [19]; Wadden et al., 2013 [21]; Sorli et al., 2017 [22]; Rubino et al., 2021 [16]; Pi-Sunyer et al., 2015 [17]; Grunberger et al., 2012 [24]; Garvey et al., 2022 [3]; and Aroda et al., 2019 [23].

The risk of bias was evaluated using the Risk of Bias 2 (RoB-2) tool (Figure 1). Disagreements were resolved by consensus or through consultation with a third reviewer. All studies were included, irrespective of their quality scores, to ensure a diverse dataset.

Statistical Analysis

Data management and cleaning were performed for all patients. Pooled descriptive analyses, including frequencies and proportions, were conducted using R version 4.4.0. Meta-analysis was conducted using the meta package. A meta-analysis of binary outcomes was performed, and the odds ratio (OR) was used as the summary statistic due to the significant diversity and heterogeneity of the studies, as well as the very low reported outcome. A fixed model was used when the tau p-value was greater than 0.1; otherwise, a random-model meta-analysis was employed.

Results

Study Selection

**Figure 2:**
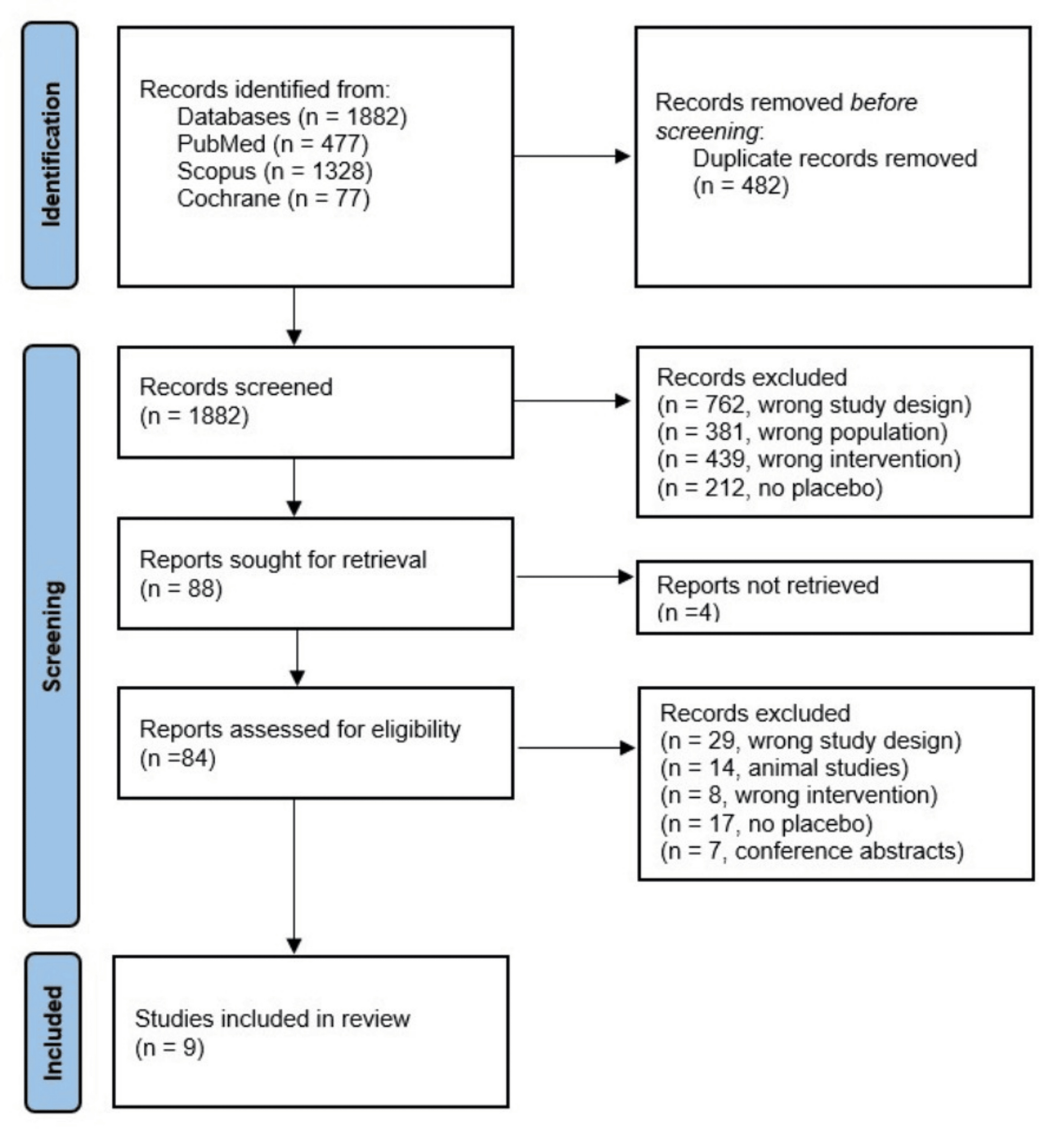
Preferred Reporting Items for Systematic Reviews and Meta-Analyses (PRISMA) flowchart.

Our database searches identified (after exclusion of 482 duplicate records) a total of 1,882 studies. After applying our predefined inclusion and exclusion criteria, nine studies (with a total of 9,078 participants) were included in the final analysis (Figure 2). Among the initial 1,882 studies, 188 were excluded because they included animal studies, case reports, reviews, irrelevant studies, incorrect medicine, flawed study design, or repetitive studies based on the title and abstract. Four studies were excluded as they could not be retrieved. For the remaining 84 studies, two authors separately read the full-text articles in detail to assess their eligibility, and 75 studies were further excluded (reviews, irrelevant studies, not reporting neoplastic events, and using other glucose-lowering agents). The remaining nine studies were included in the final analysis.

Study Characteristics

The nine studies included in the final analysis were published between 2012 and 2022 (Table 1), all of which were RCTs, and the duration of the studies ranged from 12 to 104 weeks. They compared treatment outcomes of GLP-1RAs intervention versus placebo (no other glucose-lowering agents/weight control medicines were used during the studies, except for some rescue medications, as needed). Six studies were on overweight/obese, non-diabetic participants, and three studies were on type 2 diabetic patients [16-21].

**Table 1:** Characteristics of included randomized controlled trials evaluating GLP-1 receptor agonists versus control (2012–2022). Design: RCT = randomized controlled trial. SC = subcutaneous. Tx/FU = treatment/follow-up duration. Values are mean (SD) unless stated. Where two numbers are shown in Tx/FU, the first is on-treatment weeks, the second is post-treatment follow-up. ¹ Rubino 2021 (STEP-4) run-in 0–20 weeks (dose escalation 16 weeks + 4 weeks maintenance), randomization 20–68 weeks; off-treatment follow-up 68–75 weeks. ² Control participants were switched to placebo during 20–68 weeks after run-in. ³ Pi-Sunyer 2015 primary analysis at 56 weeks (+2 weeks safety); subsequent discontinuation/extension phases not included in this synthesis; pre-diabetes extension to 160 weeks (ClinicalTrials.gov NCT01272219). ⁴ “Total N randomized” vs. “exposed” differs; 3,723 randomized; safety population exposed to ≥1 dose = 3,731 (see trial reporting). ⁵ Some randomized participants were not exposed to the study drug. ⁶ Grunberger 2012: 2-week screening; 4–8-week lead-in (8-week metformin washout) before 12-week treatment and 4-week safety follow-up. ⁷ Wadden 2013: eligibility required ≥5% weight loss during a 4–12-week low-calorie diet run-in; then 56-week treatment and 12-week follow-up. ⁸ Baseline values reported at post–run-in randomization.

Study (year) [ref]	Design	Sex, female/male (n)	Mean age, y (SD)	Weight, kg (SD)	BMI, kg/m² (SD)	HbA1c%, mean (SD)	Tx/FU, weeks	Total N randomized	GLP-1RA arm, n	Control arm, n	Intervention
Rubino et al., 2021 [16]	RCT	634/169	46 (12)	96.1 (22.6)	34.4 (7.0)	5.4 (0.3)	68/7¹	803	535	268²	Semaglutide 2.4 mg SC weekly
Sorli et al., 2017 [22]	RCT	177/210	53.7 (11.3)	91.9 (23.8)	32.93 (7.68)	8.05 (0.85)	30/5	387	258	129	Semaglutide 0.5 mg or 1 mg SC weekly
Pi-Sunyer et al., 2015 [17]	RCT	2928⁵/803⁵	45.1 (12.0)	106.2 (21.4)	38.3 (6.4)	5.6 (0.4)	56³	3,723⁴	2,481	1,242	Liraglutide 3.0 mg SC daily
Aroda et al., 2019 [23]	RCT	346/357	55.0 (11.0)	88.1 (22.1)	31.8 (6.6)	8.0 (0.7)	26/5	703	525	178	Oral semaglutide 3, 7, or 14 mg daily
Wilding et al., 2021 [19]	RCT	1453/508	46.0 (13)	105.3 (21.9)	37.9 (6.7)	5.7 (0.3)	68/7	1,961	1,306	655	Semaglutide 2.4 mg SC weekly
Wadden et al., 2021 [20]	RCT	495/116	46 (13)	105.8 (22.9)	38.0 (6.7)	5.7 (0.3)	68/7	611	407	204	Semaglutide 2.4 mg SC weekly
Garvey et al., 2022 [3]	RCT	236/68	47.3 (11.0)	106.0 (22.0)	38.5 (6.9)	5.7 (0.4)	104/7	304	152	152	Semaglutide 2.4 mg SC weekly
Grunberger et al., 2012 [24]	RCT	90/74	56.6 (8.8)	88.2 (18.6)	32.1 (4.8)	7.2 (0.6)	12/4⁶	164	132	32	Dulaglutide 0.1, 0.5, 1.0, 1.5 mg SC weekly
Wadden 2013 [21]	RCT	343/79	46.2 (11.5)⁸	99.6 (21.0)⁸	35.6 (5.9)⁸	5.6 (0.4)⁸	56/12⁷	422	212	210	Liraglutide 3.0 mg SC daily

The total number of participants across the nine included studies was 9,078, with approximately 6,008 participants exposed to GLP-1RAs intervention and 3,070 exposed to placebo. Most participants were female (n = 6,702). The mean age of the participants was 46.9 (SD = 3.2) years, the mean weight was 102.5 (SD = 6.1) kg, the mean body mass index (BMI) was 36.9 (SD = 2.2) kg/m², and the mean HbA1c was 5.9% (SD = 0.8).

GLP-1RAs in Overweight or Obese Participants

The demographics and baseline characteristics were similar between these six studies (Table 1). The mean age was 45.6 (SD = 0.6) years, the mean body weight was 104.5 (SD = 3.2) kg, the mean BMI was 37.6 (SD = 1.3) kg /m2, and the mean HbA1c was 5.6 (SD = 0.1).

The Rubino et al. (2021) STEP-4 withdrawal trial was conducted at 73 sites in 10 countries to compare the effect of continuing once-weekly treatment with semaglutide, 2.4 mg, versus switching to placebo (both with lifestyle intervention) on body weight in participants with overweight/obesity who reached a semaglutide treatment dosage of 2.4 mg once weekly during an initial 20-week run-in, and were randomized to continued treatment with semaglutide versus placebo (switch to placebo) for an additional 48 weeks. At the end of this period, a significant difference in weight change was observed between the intervention and placebo groups [16].

Pi-Sunyer et al. conducted a study at 191 sites in 27 countries in Europe, North America, South America, Asia, Africa, and Australia [17]. This clinical trial aimed to evaluate the potential of liraglutide to induce and maintain weight loss over 56 weeks in obese subjects or overweight comorbidities (this is the main study period included in our current study), and to investigate the long-term potential of liraglutide to delay the onset of type 2 diabetes in subjects diagnosed with pre-diabetes at baseline. Based on BMI and pre-diabetes status, subjects were randomized to either 68 weeks (56 weeks of randomized treatment followed by a 12-week re-randomized treatment period) or 160 weeks of treatment (only applicable to subjects with pre-diabetes status at baseline). Our current review does not include these additional periods of 12 weeks and 160 weeks. In this study, 3.0 mg of liraglutide, as an adjunct to diet and exercise, was associated with reduced body weight and improved metabolic control [18]. These additional periods (12 weeks and 160 weeks) are not included in the current study. In this study, 3.0 mg of liraglutide, as an adjunct to diet and exercise, was associated with reduced body weight and improved metabolic control [17].

Wilding et al. found that 68 weeks of treatment with once-weekly semaglutide (at a dose of 2.4 mg) or a placebo plus lifestyle intervention was effective. The trial was conducted at 129 sites in 16 countries across Asia, Europe, North America, and South America, involving participants who were overweight or had obesity. Semaglutide, administered once weekly in combination with lifestyle intervention, was associated with a sustained, clinically relevant reduction in body weight [19].

Wadden et al. conducted a study at 41 sites in the United States. This randomized clinical trial included overweight or obese adults who underwent 68 weeks of treatment with once-weekly semaglutide versus placebo, combined with intensive behavioral therapy (and a low-calorie diet for the initial eight weeks). A statistically significant reduction was noted in body weight with semaglutide compared with placebo [20].

Garvey et al. conducted a study at 41 sites across five countries (Canada, Italy, Hungary, Spain, and the United States). Treatment with once-weekly semaglutide in conjunction with behavioral intervention in overweight adults (with at least one comorbidity) or obesity (without diabetes) was associated with clinically ﻿﻿impactful and sustained weight loss at week 104, along with improvements in weight-related cardiometabolic risk factors [3].

Wadden et al. conducted a study at 26 sites in the United States and 10 in Canada. A 56-week randomized, double-masked, placebo-controlled trial examined the efficacy of liraglutide in maintaining prior weight loss achieved with a low-calorie diet for a 12-week run-in period (from week -12 to week 0), followed by a 56-week main trial period (weeks 0-56) and a 12-week follow-up period (weeks 56-68). This clinical trial aimed to evaluate the potential of liraglutide in maintaining long-term weight loss in obese non-diabetic subjects, as well as in overweight subjects with medical problems such as hypertension (high blood pressure) or dyslipidemia [21]. Therefore, with diet and exercise, liraglutide maintained the weight loss achieved by caloric restriction and induced further weight loss over 56 weeks. Improvements in some cardiovascular risk factors were also observed.

GLP-1RAs in Patients With Diabetes

Patients with type 2 diabetes who were treated naively with only diet and exercise alone or treated with lifestyle measures with metformin, which was discontinued and washed out before randomization. The demographics and baseline characteristics of the three studies were similar (Table 1). The mean age was 54.8 (SD = 0.9) years, the mean body weight was 89.3 (SD = 1.8) kg, the mean BMI was 32.2 (SD = 0.5) kg/m², and the mean HbA1c was 7.9 (SD = 0.3).

Sorli et al. (2017) [22] conducted a study at 72 sites in Canada, Italy, Japan, Mexico, Russia, South Africa, the United Kingdom, and the United States. The study assessed the efficacy, safety, and tolerability of semaglutide monotherapy (0.5 mg/1.0 mg semaglutide) with placebo in treatment-naive patients with type 2 diabetes who had insufficient glycemic control with diet and exercise alone. Semaglutide significantly improved HbA1c levels and body weight in patients with type 2 diabetes compared with placebo, and showed a similar safety profile to that of currently available GLP-1RAs.

Aroda et al. (2019) [23] conducted a study at 93 sites across nine countries (Algeria, Bulgaria, the Czech Republic, Japan, Mexico, Russia, Serbia, Turkey, and the United States). They compared the efficacy of semaglutide as monotherapy with placebo in patients with type 2 diabetes managed by diet and exercise alone. Oral semaglutide monotherapy demonstrated superior and clinically relevant improvements in HbA1c (all doses) and body weight loss (14 mg dose) compared to placebo, with a safety profile consistent with other GLP-1RAs.

Grunberger et al. (2012) [24] conducted a study at 44 sites in seven countries, aiming to evaluate dose-dependent effects and to assess the safety and efficacy of once-weekly dulaglutide (0.1, 0.5, 1.0, and 1.5 mg dulaglutide) on glycemic control in patients with type 2 diabetes treated with lifestyle measures, who were anti-hyperglycemic medication-naive or who had discontinued metformin monotherapy (eight-week washout after discontinuing metformin before randomization). The study showed a significant dose-dependent reduction in HbA1c; the reduction in the 0.5, 1.0, and 1.5 mg dulaglutide groups was greater than in the placebo group. Dose-dependent weight loss was observed across all doses, but none of the groups showed a significant difference from the placebo. In this study, dulaglutide displayed an acceptable safety and tolerability profile.

Liraglutide

Our review included two trials in which participants were exposed to 3 mg of once-daily liraglutide. The first trial, Wadden et al., conducted on 422 patients, revealed that, compared to baseline, the body weight of the intervention group (n = 212) decreased by 7.2%, whereas the placebo group (n = 210) decreased by 7%. Both groups showed similar reductions in HbA1c of 0.3% (Table 2).

**Table 2:** Experimental and control group characteristics and neoplasm status for each participant. MBWR = mean body weight reduction

Study	Drug	Group	Sample size	MBWR (%)	Mean HbA1c reduction (%)	Number of neoplasms	Types of neoplasms
Wadden et al., 2013 [13]	Liraglutide	Intervention	212	7.2	0.3	12	Benign: breast neoplasm, fibroma, lipoma, papilloma, prolactinoma, uterine leiomyoma. Malignant: thyroid (3), breast (2), and ovarian (1)
		Placebo	210	7	0.3	4	Benign: pseudolymphoma and benign skin papilloma. Malignant: basal cell carcinoma and lung metastasis
Pi-Sunyer et al., 2015 [9]	Liraglutide	Intervention	2,481	8 ± 6.7	0.3 ± 0.28	4	Breast cancer (4)
		Placebo	1,242	2.6 ± 5.7	0.06 ± 0.3	1	Breast cancer (1)
Garvey et al., 2022 [12]	Semaglutide	Intervention	152	15.2% ± 0.9%	0.4% ± 0.03%	2	Basal cell carcinoma and Bowen’s disease
		Placebo	152	2.6% ± 1.1%	0.1% ± 0.03%	4	Breast cancer (2) and lung cancer (2)
Sorli et al., 2017 [14]	Semaglutide	Intervention	0.5 mg: 128 1 mg: 130	0.5 mg: 4.15, 1 mg: 4.68	0.5 mg: 1.45%, 1 mg: 1.55%	0.5 mg: 4, 1 mg: 5	Breast cancer, prostate cancer, basal cell carcinoma, and squamous cell carcinoma of the skin
		Placebo	129	1.1	0.02%	0	None
Wadden et al., 2021 [11]	Semaglutide	Intervention	407	0.51	16	3	Basal cell carcinoma, breast cancer, and thyroid cancer
		Placebo	204	0.27	5.7	1	Breast cancer
Aroda et al., 2019 [15]	Semaglutide	Intervention	525	-1.96 (3 mg), -2.81 (7 mg), -4.65 (14 mg)	-0.8 (3 mg), -1.3 (7 mg), -1.5 (14 mg)	6	Papillary thyroid cancer
		Placebo	178	1.69	-0.1	9	Not specified
Wilding et al., 2021 [10]	Semaglutide	Intervention	407	-14.85	-0.45	14	Not specified
		Placebo	204	-2.4	-0.15	7	Not specified
Rubino et al., 2021 [8]	Semaglutide	Intervention	535	-10.6 (at week 0–20), -7.9 (at week 20–68)	-0.4 (at week 0–20), -0.1 (at week 20–68)	6	Breast neoplasm, endometrial adenocarcinoma, lymphoma, and melanoma
		Placebo	268	-10.6 (at week 0-20), +6.9 (at week 20–68)	-0.4 (at week 0–20), 0.1 (at week 20–68)	1	Lung cancer
Grunberger et al., 2012 [24]	Dulaglutide	Intervention	35 (0.1 mg) 34 (0.5 mg) 34 (1.0 mg) 29 (1.5 mg)	-0.23 (0.1 mg), -0.33 (0.5 mg), -1.27 (1.0 mg), -1.75 (1.5 mg)	-0.37 (0.1 mg), -0.89 (0.5 mg), -1.04 (1.0 mg), -1.04 (1.5 mg)	1	Breast cancer (with dulaglutide 0.5 mg)
		Placebo	32	-1.54	-0.01	0	-

The second study, Pi-Sunyer et al., which included 2,481 participants in the intervention group and 1,242 in the placebo group, showed more variation between groups. Body weight decreased by 8% ± 6.7% in the intervention group and by 2.6% ± 5.7% in the placebo group. In this second study, HbA1c levels decreased by 0.3% ± 0.28% in the intervention group and by 0.06% ± 0.3 in the placebo group (Table 2). Regarding the neoplasm incidence, Pi-Sunyer et al. revealed five cases of breast cancer, with four in the intervention group and only one in the placebo group. No other types of malignancies were observed in this study. In the study by Wadden et al., the intervention group included 12 neoplasms, comprising six benign and six malignant cases, while the placebo group had four neoplasms, consisting of two benign and two malignant cases. Refer to Table 2 for full details on neoplasms.

**Figure 3:**
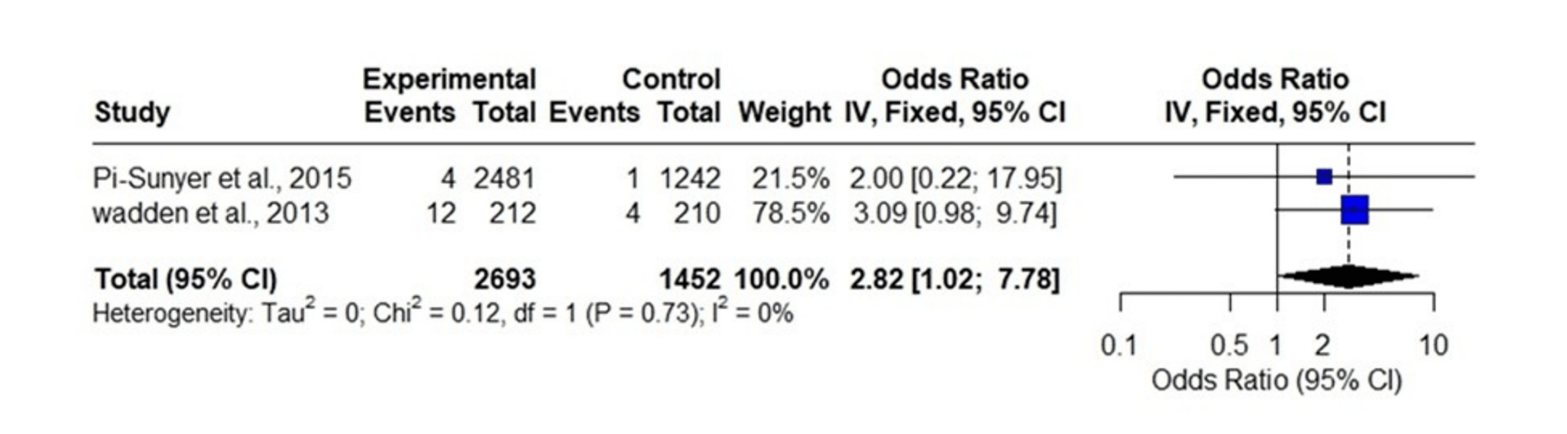
Forest plot of the incidence of neoplasms with the use of liraglutide. References: Wadden et al., 2013 [21]; Pi-Sunyer et al., 2015 [17].

The meta-analysis of liraglutide included two studies (Figure 3), with Pi-Sunyer et al. (2015) reporting an OR of 2.0040 (95% confidence interval (CI) = 0.2238 to 17.9489) and Wadden et al. (2013) reporting an OR of 3.0900 (95% CI = 0.9802 to 9.7412). The total number of observations was 4,145, with 2,693 in the experimental group and 1,452 in the control group, resulting in a total of 21 events. The fixed-effect model yielded an overall OR of 2.8150 (95% CI = 1.0180 to 7.7843), with a p-value of 0.0461, indicating a statistically significant effect.

Semaglutide

Our study included six trials involving the drug semaglutide (Table 2). The first trial, Garvey et al., included 152 participants in the intervention and placebo groups. The intervention group experienced a weight reduction of 15.2% ± 0.9%, while the placebo group saw a reduction of 2.6% ± 1.1%. HbA1c levels decreased by 0.4% ± 0.03% in the intervention group and by 0.1% ± 0.03% in the placebo group. Regarding neoplasm incidence, the study reported two cases in the intervention group and four cases in the placebo group.

The second trial, conducted by Sorli et al., involved 387 participants. The intervention group was divided into two subgroups: 128 participants received semaglutide at a dose of 0.5 mg, and 130 received 1 mg. The placebo group consisted of 129 participants. In the 0.5 mg group, body weight decreased by 3.73 kg (4.15%); in the 1 mg group, it decreased by 4.53 kg (4.68%). The placebo group saw a reduction of 0.98 kg (1.1%). HbA1c levels declined by 1.45% in the 0.5 mg group, 1.55% in the 1 mg group, and 0.02% in the placebo group. The intervention group, of semaglutide, had four cases (two malignant and two benign), while the group receiving 1 mg had five cases (two malignant and three benign). No neoplasms were observed in the placebo group.

In a study by Wadden et al. (2021), semaglutide at a target dose of 2.4 mg was evaluated over 68 weeks, followed by a seven-week follow-up period. Participants who could not tolerate the 2.4 mg dose were adjusted to 1.7 mg and encouraged to re-escalate to 2.4 mg if tolerated. The study included 407 participants on semaglutide and 204 on placebo. The reduction in HbA1c was -0.51% in the semaglutide group and -0.27% in the placebo group. The mean body weight reduction was -16.8 kg with semaglutide and -6.2 kg (-5.7%) with placebo. Neoplasm events included three cases (basal cell carcinoma, breast cancer, and thyroid cancer) in the semaglutide group and one breast cancer case in the placebo group.

Aroda et al. evaluated three doses of semaglutide (3 mg, 7 mg, and 14 mg) over 26 weeks. Among the 525 participants receiving semaglutide and 178 receiving placebo, the 14 mg dose produced the highest HbA1c reduction of 1.5% and a body weight reduction of 4.1 kg. Six neoplasm events occurred in the intervention group, including one papillary thyroid cancer case, and nine neoplasm cases occurred in the placebo group, three of which were malignant.

Wilding et al. assessed once-weekly semaglutide (2.4 mg) over 68 weeks, with 1,306 participants on semaglutide and 655 on placebo. The HbA1c reduction was -0.45% with semaglutide and -0.15% with placebo. Mean body weight reduction was 15.3 kg with semaglutide, compared to 2.6 kg (-2.41%) with placebo. The semaglutide group reported 14 neoplasm events, while the placebo group reported seven events.

Rubino et al. studied semaglutide over 68 weeks, starting with 20 weeks of increasing doses to 2.4 mg weekly, followed by 48 weeks at a dose of 2.4 mg per week. The placebo group mirrored the initial dosing schedule before switching to placebo. The study involved 535 participants on semaglutide and 268 on placebo. HbA1c reduction in the semaglutide group was -0.4% ± 0.2% initially and -0.1 (-0.2 to -0.1) later, compared to -0.4% ± 0.2% and 0.1 (0.1 to 0.1) in the placebo group. At 20 weeks, both groups had a 10.6% body weight reduction; the semaglutide group, treated with semaglutide, resulted in a 7.9% decrease (from 7.2% to 8.6%), while the placebo group experienced a 6.9% increase (from 5.8% to 7.9%). Neoplasm events included six cases in the semaglutide group (three breast neoplasms) and one metastatic lung cancer case in the placebo group.

**Figure 4:**
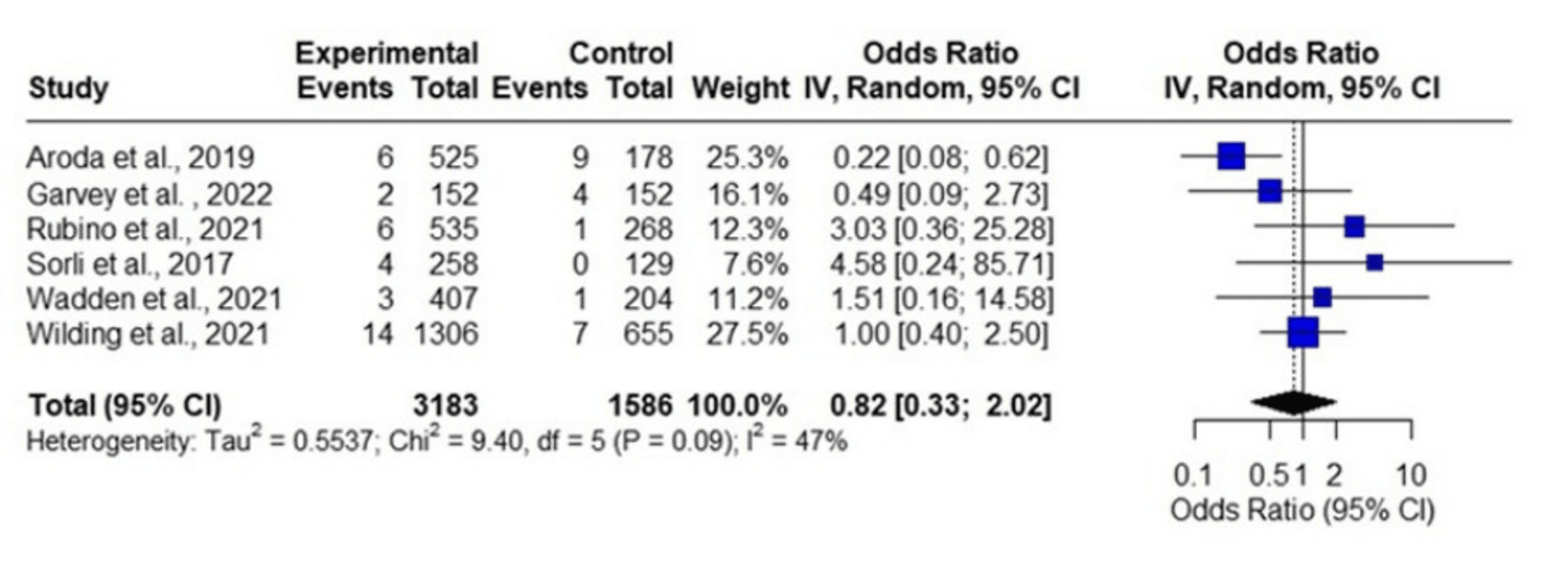
Forest plot of meta-analysis of the incidence of neoplasms with the use of semaglutide. References: Wilding et al., 2021 [19]; Wadden et al., 2013 [21]; Sorli et al., 2017 [22]; Rubino et al., 2021 [16]; Garvey et al., 2022 [3]; and Aroda et al., 2019 [23].

The meta-analysis of semaglutide included six studies (Figure 4). The total number of observations was 4769, with 3,183 in the experimental group and 1,586 in the control group, and 57 events. The random-effects model yielded an overall OR of 0.8179 (95% CI = 0.3316 to 2.0169), with a p-value of 0.6624, indicating a non-significant effect.

Dulaglutide

In a study by Grunberger et al., the effects of various doses of dulaglutide on HbA1c and body weight were investigated over a 12-week therapy period (Table 2). The administered doses were 0.1 mg, 0.5 mg, 1.0 mg, and 1.5 mg, with 35, 34, 34, and 29 participants receiving each dose, respectively. Additionally, 32 patients received a placebo. HbA1c reductions were -0.37% for the 0.1 mg dose, -0.89% for the 0.5 mg dose, -1.04% for both the 1.0 mg and 1.5 mg doses, and -0.01% for the placebo group. Mean body weight reductions were -0.2 ± 0.4 kg for the 0.1 mg dose, -0.3 ± 0.4 kg for the 0.5 mg dose, -1.1 ± 0.4 kg for the 1.0 mg dose, -1.5 ± 0.5 kg for the 1.5 mg dose, and -1.4 ± 0.5 kg for the placebo. Regarding neoplasm events, one case of breast cancer was reported in the group receiving the 0.5 mg dose, whereas no neoplasms were reported in the placebo group.

Incidence of Neoplasia With All GLP-1RAs

**Figure 5:**
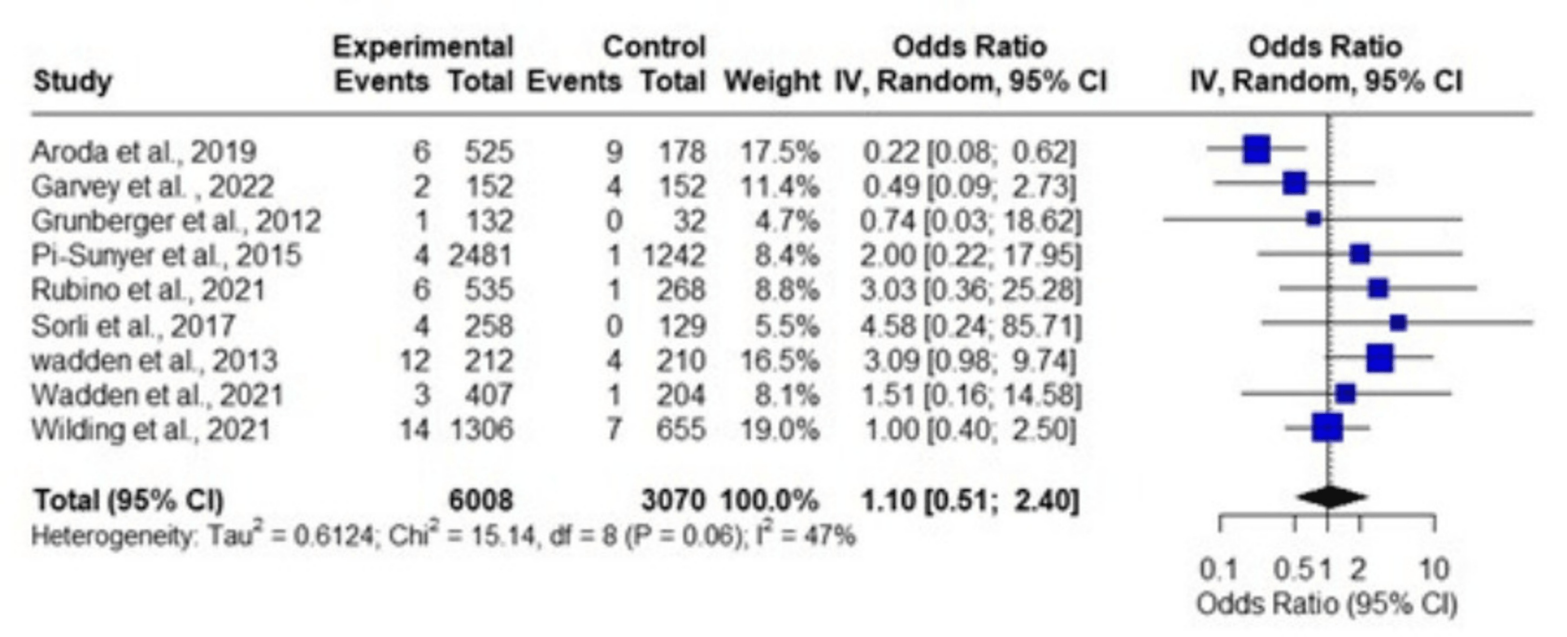
Meta-analysis of the incidence of neoplasms with the use of semaglutide, liraglutide, and dulaglutide. References: Wilding et al., 2021 [19]; Wadden et al., 2021 [20]; Wadden et al., 2013 [21]; Sorli et al., 2017 [22]; Rubino et al., 2021 [16]; Pi-Sunyer et al., 2015 [17]; Grunberger et al., 2012 [24]; Garvey et al., 2022 [3]; and Aroda et al., 2019 [23].

The meta-analysis encompassing liraglutide, semaglutide, and dulaglutide included nine studies (Figure 5). The total number of observations was 9,078, with 6,008 in the experimental group and 3,070 in the control group, and a total of 79 events. The random-effects model yielded an overall OR of 1.1022 (95% CI = 0.5065 to 2.3983), with a p-value of 0.8063, indicating a non-significant effect.

Discussion

This meta-analysis investigated the correlation between the use of GLP-1RAs and the risk of mediating neoplasms and malignancies in diabetic and non-diabetic overweight or obese patients, compared to placebo. The analysis incorporated nine RCTs published between 2012 and 2022, encompassing a total of 9,078 participants. Among these, six trials enrolled overweight/obese non-diabetic individuals, while the remaining three focused on type 2 diabetic patients. The trial durations ranged from 12 to 104 weeks.

The nine RCTs assessed three GLP-1RAs: liraglutide (3 mg once daily in two studies), semaglutide (in six studies), and dulaglutide (in one study at doses of 0.1 mg, 0.5 mg, 1.0 mg, and 1.5 mg). Across the studies, 79 neoplasm events were reported. Of these, 21 occurred in liraglutide studies, 57 in semaglutide studies, and one in the dulaglutide study. Benign neoplasms included breast neoplasms, fibroma, lipoma, papilloma, prolactinoma, uterine leiomyoma, pseudolymphoma, and benign skin papilloma (Table 2). Malignant neoplasms reported across the studies included thyroid cancer, breast cancer, ovarian cancer, basal cell carcinoma, lung metastasis, lung cancer, Bowen’s disease, prostate cancer, squamous cell carcinoma of the skin, papillary thyroid cancer, endometrial adenocarcinoma, lymphoma, and melanoma.

The limited duration of follow-up in the included studies raises concerns about the validity of cancer risk assessment. Tumor development is generally a slow process, often requiring years to manifest. For example, one study by Grunberger et al. [24] had only 12 weeks of treatment and a four-week follow-up, which is insufficient to establish a causal relationship between GLP-1RA exposure and cancer development. Friberg et al. [25] similarly highlighted that breast, prostate, and colon cancers often exhibit long tumor volume doubling times, usually spanning months to years. Collins et al. [26] were among the first to observe the growth rates of pulmonary metastases, concluding that tumor growth: (1) remains constant over extended periods, (2) is frequently slow, and (3) varies by histological type. These findings indicate that cancers diagnosed within short follow-up periods are unlikely to be causally related to GLP-1RA interventions [24].

The broader evidence base on GLP-1RAs and cancer presents a complex and somewhat conflicting landscape. Selverii et al. [27] reported a moderate increase in the risk of thyroid cancer associated with GLP-1RA use. Elashoff et al. [28], in a population-based study using the U.S. FDA’s Adverse Event Reporting System, found significantly higher reports of pancreatic cancer among patients using sitagliptin or exenatide, though these data were hypothesis-generating. In contrast, Liu et al. [29] concluded in a 2019 meta-analysis that current evidence does not strongly support an elevated cancer risk with GLP-1RA use in T2DM patients. Similarly, Piccoli et al. [30] found no increased risk of breast cancer or benign neoplasms. Knapen et al. [31], in a 2015 meta-analysis, reported no association between the use of incretins and the risk of pancreatic cancer.

The average age of participants in this meta-analysis was 46.9 years, a relatively young cohort with a lower baseline cancer risk. As cancer incidence generally increases with age, this demographic may underestimate the long-term risks associated with GLP-1RA exposure. The younger age group, combined with short trial durations, limits the ability to fully evaluate cancer risk over time.

While many meta-analyses and RCTs suggest no significant risk, caution remains warranted due to limitations in study design and population heterogeneity. GLP-1RAs have become increasingly popular in the management of type 2 diabetes and obesity, raising continued concerns about their long-term safety profile. Several preclinical studies suggest a potentially protective role of GLP-1RAs in cancer biology. For instance, exendin-4 was shown in molecular studies to inhibit prostate tumor growth by suppressing ERK-MAPK activation [32]. Iwaya et al. [33] found similar anti-tumor effects in breast cancer, reporting that GLP-1RAs inhibited NF-κB activation. Both Nomiyama et al. and Iwaya et al. detected GLP-1R expression in human prostate and breast cancer tissues, respectively [32,33].

Thyroid cancer risk presents another area of uncertainty. Thyroid malignancies originate from distinct cell types, follicular cells (e.g., papillary thyroid carcinoma) and parafollicular C cells (e.g., medullary carcinoma), each with unique biological features [34]. Both thyrocytes and C cells express GLP-1 receptors [35], and GLP-1 receptor expression is higher in PTC cells than in normal thyroid tissue [36,37]. While Selverii et al. [27] reported a moderate risk increase, further data are required to evaluate the biological plausibility and clinical significance across thyroid cancer subtypes.

Several studies have also explored the relationship between GLP-1RAs and hepatocellular carcinoma (HCC). These studies generally suggest favorable outcomes. GLP-1RAs appear to regulate molecular pathways involved in inflammation, tumor cell proliferation, and oxidative stress. Zhou et al. [38] demonstrated that Ex-4 inhibited HCC progression through the cAMP-PKA-EGFR-STAT3 signaling axis. Zhu et al. [39] found that GLP-1RAs improved intrahepatic adiposity, subcutaneous and visceral adipose tissue profiles, inflammatory markers, glycemic indices, and serum lipids in patients with type 2 diabetes and non-alcoholic fatty liver disease.

Nonetheless, GLP-1RAs are associated with specific adverse effects. Gastrointestinal disturbances, especially nausea, are most common, followed by headache, nasopharyngitis, and injection site reactions. Although some case reports have described acute kidney injury, particularly with exenatide, they were primarily linked to dehydration from gastrointestinal side effects. Importantly, there is no current evidence of adverse cardiovascular effects in T2DM patients using GLP-1RAs [40-49].

Although RCTs were prioritized in this meta-analysis due to their methodological robustness, excluding observational data limits insight into long-term outcomes. Observational studies have yielded mixed results. A systematic review of 45 clinical trials showed a 28% increased risk of thyroid disorders among GLP-1RA users [49]. A nested case-control study using the French SNDS database found a higher risk of thyroid cancer (HR = 1.58, 95% CI = 1.27-1.95) among patients with T2DM who used GLP-1RAs [43]. In contrast, an extensive cohort study from Scandinavia with a mean follow-up of 3.9 years found no substantial increase in thyroid cancer risk. The U.S. FDA’s Adverse Event Reporting System data also reported significantly elevated proportional reporting ratios (PRRs) for thyroid cancer (PRR = 27.43) and papillary thyroid cancer (PRR = 8.68) associated with GLP-1RA and DPP-4 inhibitor use [50,51]. Given that GLP-1 receptors are expressed in thyroid C-cell hyperplasia and neoplasia [52], the long-term implications of enhanced receptor signaling merit careful monitoring.

Concerns about pancreatic cancer remain unresolved. While Elashoff et al. [28] reported increased reporting of pancreatic cancer with sitagliptin and exenatide, other studies have not corroborated this finding. Several studies found no increased risk of pancreatic cancer associated with GLP-1RA treatment, underscoring the inconclusive nature of the available data.

This meta-analysis included primarily semaglutide trials, followed by liraglutide and dulaglutide. The primary efficacy outcomes were reductions in body weight and HbA1c levels, while neoplasms were reported as adverse events. Studies such as those by Wadden et al. [21] and Pi-Sunyer et al. [17] confirmed significant weight loss and glycemic improvements, though neoplasm incidence varied. The meta-analysis revealed moderate heterogeneity (I² = 46.8%), likely due to differences in patient populations, study durations, and GLP-1RA formulations.

While the included trials were double-blinded and robust, variability in sample size and inconsistent adjustment for confounders may have affected reliability. Selection and performance biases were unlikely, but reporting and detection biases could not be ruled out. Notably, the mean BMI across participants was 36.9 kg/m², categorizing most as obese. As obesity is a known risk factor for cancers such as colorectal and breast cancer, this confounding variable complicates attribution of cancer outcomes solely to GLP-1RA exposure.

Several key limitations should be taken into account when interpreting these findings. The relatively short duration of most trials does not allow for adequate assessment of cancer development. Participants in RCTs tend to be healthier than real-world patients, which could result in an underestimation of risks. Additionally, the occurrence of neoplasms was neither a primary nor a secondary endpoint in most trials, raising the possibility of reporting bias. The use of rescue medications, often permitted in trial protocols, further complicates the interpretation of outcomes. Many trials also failed to provide detailed stratification by obesity status, limiting insights into differential risks between obese and non-obese patients [46-53].

To overcome these limitations, future studies should extend follow-up periods, include diverse populations, and systematically monitor cancer outcomes to gain a more comprehensive understanding of the topic. Cancer endpoints should be explicitly defined as primary or secondary outcomes to ensure consistent reporting. Subgroup analyses accounting for rescue medication use and baseline characteristics are necessary to disentangle confounding effects.

One of the most significant limitations of this meta-analysis is the low number of neoplasm events, leading to wide CIs and reduced precision. Consequently, neither a carcinogenic nor a protective effect of GLP-1RAs can be definitively established. Larger, longer-term studies are essential to refine these risk estimates.

## Conclusions

This meta-analysis confirmed that GLP-1RAs, specifically semaglutide, liraglutide, and dulaglutide, are effective in lowering HbA1c and body weight in patients with type 2 diabetes and obesity. However, due to short study durations and moderate heterogeneity, definitive conclusions about long-term cancer risk cannot be made. While no strong link to neoplasms was found, cautious interpretation is needed. Differences in treatment response between obese and normal-weight individuals may introduce confounding. Clinicians should personalize treatment decisions and provide patients with information about the benefits and risks. Future studies should include longer follow-up and stratified analyses to better assess the long-term safety of GLP-1RAs and their differential effects across patient populations.
